# Characterization of the inositol monophosphatase gene family in Arabidopsis

**DOI:** 10.3389/fpls.2014.00725

**Published:** 2015-01-09

**Authors:** Aida Nourbakhsh, Eva Collakova, Glenda E. Gillaspy

**Affiliations:** ^1^Department of Human and Molecular Genetics, Virginia Commonwealth UniversityRichmond, VA, USA; ^2^Department of Plant Pathology, Physiology, and Weed Science, Virginia Polytechnic Institute and State UniversityBlacksburg, VA, USA; ^3^Department of Biochemistry, Virginia Polytechnic Institute and State UniversityBlacksburg, VA, USA

**Keywords:** histidine, inositol, histidinol phosphatase, inositol monophosphatase, IMPL2

## Abstract

Synthesis of *myo*-inositol is crucial in multicellular eukaryotes for production of phosphatidylinositol and inositol phosphate signaling molecules. The *myo*-inositol monophosphatase (IMP) enzyme is required for the synthesis of *myo*-inositol, breakdown of inositol (1,4,5)-trisphosphate, a second messenger involved in Ca^2+^ signaling, and synthesis of L-galactose, a precursor of ascorbic acid. Two *myo*-inositol monophosphatase -like (IMPL) genes in Arabidopsis encode chloroplast proteins with homology to the prokaryotic IMPs and one of these, IMPL2, can complement a bacterial histidinol 1-phosphate phosphatase mutant defective in histidine synthesis, indicating an important role for IMPL2 in amino acid synthesis. To delineate how this small gene family functions in inositol synthesis and metabolism, we sought to compare recombinant enzyme activities, expression patterns, and impact of genetic loss-of-function mutations for each. Our data show that purified IMPL2 protein is an active histidinol-phosphate phosphatase enzyme in contrast to the IMPL1 enzyme, which has the ability to hydrolyze D-galactose 1-phosphate, and D-*myo*-inositol 1-phosphate, a breakdown product of D-inositol (1,4,5) trisphosphate. Expression studies indicated that all three genes are expressed in multiple tissues, however, IMPL1 expression is restricted to above-ground tissues only. Identification and characterization of *impl1* and *impl2* mutants revealed no viable mutants for IMPL1, while two different *impl2* mutants were identified and shown to be severely compromised in growth, which can be rescued by histidine. Analyses of metabolite levels in *impl2* and complemented mutants reveals *impl2* mutant growth is impacted by alterations in the histidine biosynthesis pathway, but does not impact *myo-inositol* synthesis. Together, these data indicate that IMPL2 functions in the histidine biosynthetic pathway, while IMP and IMPL1 catalyze the hydrolysis of inositol- and galactose-phosphates in the plant cell.

## Introduction

The *myo*-inositol (inositol) synthesis pathway is crucial in many multicellular eukaryotes for the production of lipid phosphatidylinositol phosphate signaling molecules (for review see Gillaspy, 2011). Inositol is also used in the synthesis of other important molecules in plants, including the glycerophosphoinositide membrane anchors, cell wall pectic non-cellulosic polysaccharides, and ascorbic acid (Loewus, 1969, 2006; Kroh et al., 1970; Chen and Loewus, 1977). Inositol monophosphatase (IMP) is a major enzyme required both for the *de novo* synthesis of inositol, and the breakdown of D-inositol (1,4,5) trisphosphate (Ins(1,4,5)P_3_) (Loewus and Loewus, 1983), a second messenger involved in many plant physiological responses (for review see Boss and Im, 2012).

We previously characterized the single, canonical IMP gene from tomato (Gillaspy et al., 1995) and Arabidopsis (Torabinejad et al., 2009), encoded by the Vitamin C 4 (VTC4; At3g02870) gene (Conklin et al., 2006). The active site of IMP has been noted to accommodate a variety of substrates, and seminal work has shown that the plant IMP can hydrolyze L-galactose 1-P (L-Gal 1-P), a precursor for ascorbic acid synthesis (Laing et al., 2004). Arabidopsis *imp* mutants have decreases in both ascorbic acid and inositol, underscoring the bifunctionality of this enzyme (Torabinejad et al., 2009). Surprisingly, *imp* mutants have only a 30% reduction in inositol content, which indicates the likely presence of other plant IMP enzymes (Torabinejad et al., 2009).

Indeed, all plants queried contain multiple IMP-like (IMPL) genes, which are closer in amino acid sequence identity to the prokaryote IMPs (Torabinejad and Gillaspy, 2006; Torabinejad et al., 2009). A *preliminary* characterization of the two Arabidopsis IMPL enzymes indicated these enzymes differ from IMP in their substrate specificity (Torabinejad et al., 2009). However, both enzymes were not stable and no kinetic characterization could be performed, precluding a definitive comparison of these enzymes to IMP (Torabinejad et al., 2009). Both IMPL1 and IMPL2 proteins have been localized to the chloroplast (Sun et al., 2009; Petersen et al., 2010), and it has been shown that heterologous expression of IMPL2 (At4g39120) but not IMPL1 (At1g31190), is sufficient to rescue the histidine auxotrophy of a *Streptomyces coelicolor* hisN mutant, which is defective in L-histidinol 1-phosphate (His 1-P) phosphatase activity (Petersen et al., 2010). This work made an important contribution to not only identifying the last missing step in histidine biosynthesis in plants, but as well suggested that either the catalytic site of IMPL2 accommodated a different substrate (i.e., His 1-P) or that IMPL2 functioned in multiple pathways (i.e., both histidine and inositol synthesis) (Petersen et al., 2010; Ingle, 2011).

Since both Arabidopsis IMPL1 and IMPL2 genes are possible candidates for a redundant IMP function, we sought to purify and characterize these enzymes. Further, given the bifunctionality of the IMP enzyme, we wanted to examine the expression patterns and impact of a loss-of-function in these genes on both the inositol and histidine synthetic pathways. Since histidine is an essential amino acid utilized for protein synthesis, a complete blockage of histidine production causes lethality in plants and leads to elevated expression of genes in other amino acid biosynthetic pathways (Guyer et al., 1995). Probably because of this, very little is known about the role of histidine in plant development and physiology. This is also influenced by the difficulty in experimentally separating the metabolic and regulatory functions of this essential amino acid and the embryo lethality that results from loss-of-function mutants of genes in the pathway (Mo et al., 2006). Indeed, *impl2* mutants have been identified previously, however embryo lethality of homozygotes limited analysis of the impact of IMPL2 mutation on plant growth and development (Petersen et al., 2010).

In this work we demonstrate kinetic analysis of recombinant AtIMPL1 and AtIMPL2 proteins and show that AtIMPL2 is uniquely able to hydrolyze His 1-P *in vitro*, while AtIMPL1 hydrolyzes D-inositol 1-phosphate (D-Ins 1-P) and D-galactose1-phosphate (D-Gal 1-P). We characterized and complemented an *impl2* mutant, and were able to grow this mutant to maturity. Thus, we were able to assess the impact of IMPL2 on histidine synthesis and show that it does not impact inositol synthesis. Interestingly, the *impl2* mutant has the described symptoms of previously reported histidine synthesis mutants such as the pale-green leaf phenotype of *agp10* (Noutoshi et al., 2005) and the root meristem defect of *hpa1* mutants (Mo et al., 2006). Thus, our biochemical and genetic data solidify the role of the IMPL2 gene in histidine synthesis in plants, and point to the IMPL1 gene as a likely candidate for regulating inositol recycling from inositol phosphate second messengers.

## Results

### Expression of recombinant IMPL1 and IMPL2 proteins

To examine the roles of IMPL1 and IMPL2 enzymes, we expressed and purified recombinant IMPL1 and IMPL2 proteins. Both genes encode putative chloroplast transit peptides, as predicted by alignment of IMPL amino acid sequences with those of non-chloroplastic IMPs. The open reading frames minus the putative chloroplastic transit peptide of the IMPL1 gene (At1g31190) and the IMPL2 gene (At4g39120) were cloned as glutathione s-transferase (GST) fusions and purified with glutathione-sepharose to greater than 95% purity as observed by SDS-PAGE (data not shown). The molecular mass of the fusion proteins is estimated to be 65 kD for IMPL1 and 60 kD for IMPL2, which is slightly larger than expected given their predicted molecular masses of 55.5 and 55.4 kD, respectively.

Because it has been shown that Mg^2+^ is necessary for maximal activity of other IMP enzymes (Gumber et al., 1984; Laing et al., 2004; Islas-Flores and Villanueva, 2007; Torabinejad et al., 2009) we delineated the optimal Mg^2+^ and pH conditions for each enzyme (Supplemental Figure 1). IMPL2 had near maximal activation at 2 mM Mg^2+^ (Supplemental Figure 1) and the concentration of Mg^2+^ in the chloroplast has been measured to be approximately 0.5 mM and to increase to approximately 2 mM in the stroma upon illumination (Ishijima et al., 2003). Therefore, for IMPL2, we used 2 mM MgCl_2_ as starting conditions to mimic the chloroplast environmental conditions during daylight. IMPL1 had slightly higher enzymatic activity at 3 mM Mg^2+^, therefore 3 mM MgCl_2_ was used in activity assays performed with IMPL1. Since IMPL1 is most active at pH 9, and IMPL2 at pH 7.5, all kinetic assays were carried out at these pH values, respectively.

**Figure 1:**
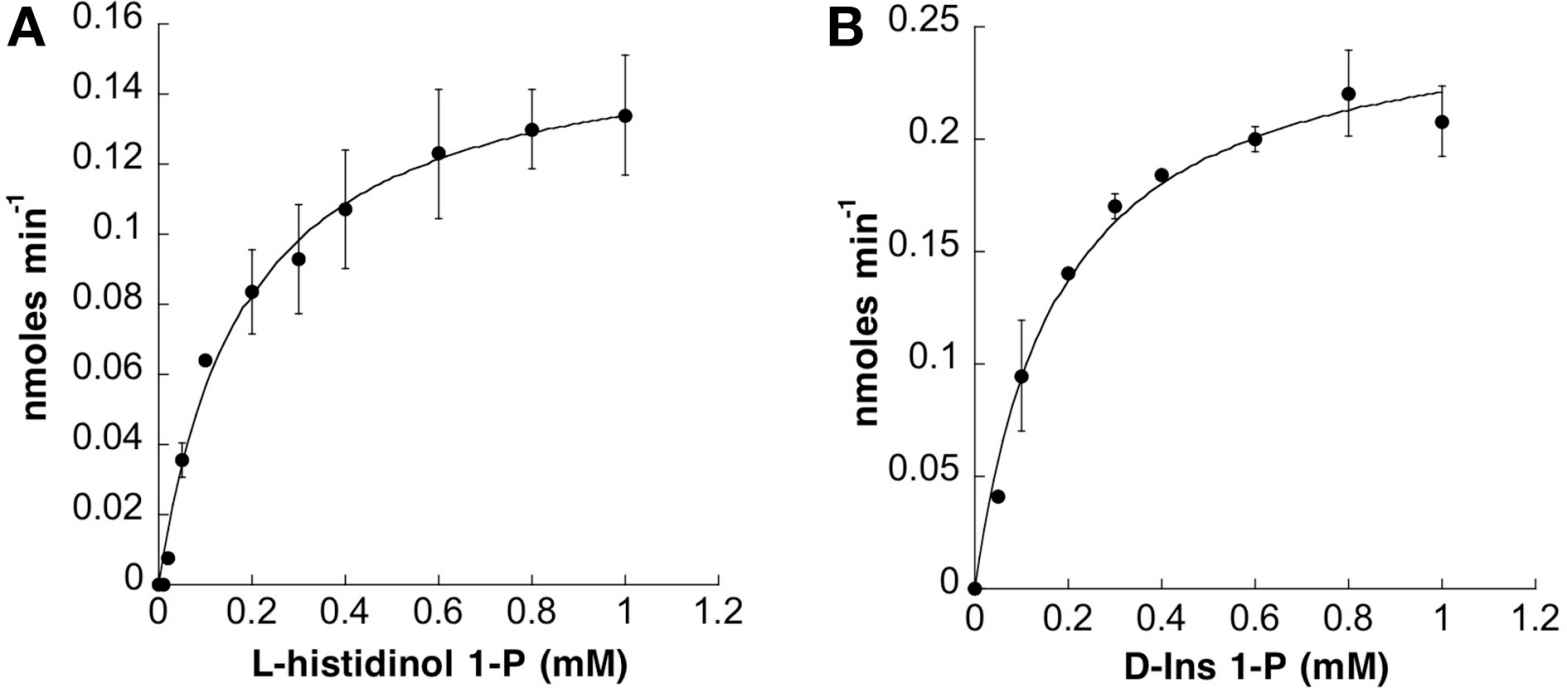
**Kinetic Analysis of IMPL1 with D-Ins 1-P and IMPL2 with Histidinol 1-P.** Phosphatase activity vs. concentration of histidinol 1-P for IMPL2 in **(A)** and D-Ins 1-P for IMPL1 in **(B)** using the reaction conditions described in “Experimental Procedures.” Data (average of 3 independent replicates) were imported into Kaleidagraph (Synergy Software) and fit to a non-linear curve based on the Michaelis-Menten equation to calculate *K_m_* and *V*_max_. The error bars represent standard deviation of the independent replicates.

Arabidopsis IMP is a bifunctional enzyme hydrolyzing L-Gal 1-P and D-inositol 3-phosphate (D-Ins 3-P) (Conklin et al., 2000; Laing et al., 2004; Torabinejad et al., 2009). It has also been reported that heterologous expression of IMPL2 was sufficient to rescue the histidine auxotrophy of a *Streptomyces coelicolor* hisN mutant (Petersen et al., 2010). Therefore, to compare the substrate preferences of IMPL enzymes, we analyzed their abilities to utilize several related substrates (Table 1). For the IMPL2 enzyme, testing of different substrates validated that IMPL2 has high specificity for His 1-P and is not able to hydrolyze D-Ins 1-P, D-Ins 3-P, L-Gal 1-P, or fructose 1,6-bisphosphate (Fru 1,6-bisP). We conclude that the IMPL2 gene encodes an active histidinol 1-P phosphatase, and is unlikely to function in inositol phosphate hydrolysis. In reaction mixtures of pH 7.5, 2 mM MgCl_2_ and 112 ng of enzyme, the *K_m_* for histidinol 1-P is 180 ± 5 μM, the *k*_cat_ is 1.3 ± 0.2 s^−1^, and the *k*_cat_/*K_m_* is 7.9 ± 0.2 × 10^3^ M^−1^s^−1^ (Figure 1 and Table 2).

**Table 1:** **Substrates tested with IMPL1**.

	**IMPL1**	**IMP**
**Substrate**	**Rate %**	**Rate %**
D-*myo*-Inositol 1-phosphate	100	100
D-Galactose 1-phosphate	105.4	16.6
β-Glycerophosphate (glycerol 2-P)	39.7	52
D-*myo*-Inositol 3-phosphate	18.8	100
D-*myo*-Inositol 2-phosphate	17.8	0.94
L-Galactose 1-phosphate	7.6	166–240
Adenosine 2′-phosphate	3.6	9.6
α-D-Glucose 1-phosphate	2.8	19.3
D-α-Glycerophosphate (glycerol 3-P)	0.24	4.9
α-D-Glucose 6-phosphate	0	0.25
D-Mannitol 1-phosphate	0	10.5
D-Sorbitol 1-phosphate	0	1.7
D-Fructose 1-phosphate	0	2.3
Fructose 1,6-bisphosphate	0	0.30
NADP	0	nd
NADPH	0	nd
PAP	0	nd
L-Histidinol 1-phosphate	0	nd
Inositol (1,4)P_2_	0	nd
Inositol (4,5)P_2_	0	nd
Inositol (1,4,5)P_3_	0	nd

*IMPL1 activity was determined at pH 9 in the presence of 3 mM MgCl_2_ using the phosphate release assay, 452 ng of IMPL1 enzyme, and 0.4 mM of the indicated substrate (substrate was present in excess amount as compared to the estimated Km value for D-myo-Inositol 1-phosphate). Reaction rates were compared with the rate of activity with 0.4 mM D-myo-Inositol 1-phosphate (units). The values for IMP enzyme were published in Torabinejad et al. (2009). nd, not determined*.

**Table 2:** **Kinetic parameters of IMPL1 and IMPL2 recombinant proteins**.

**Enzyme (Substrate)**	***K*_**m**_ (μM)**	***k*_**cat**_ (s^−**1**^)**	***k*_**cat**_/*K*_**m**_ (s^−**1**^M^−**1**^)**
IMPL2 (Histidinol 1-phosphate)	180 ± 5	1.3 ± 0.2	7.9 ± 0.2 × 10^3^
IMPL1 (D-*myo*-Inositol 1-phosphate)	180 ± 3	0.6 ± 0.1	3.3 ± 0.1 × 10^3^
IMPL1 (D-Galactose 1-phosphate)	450 ± 60	2.4 ± 1.3	5.3 ± 0.5 × 10^3^

*The initial rate for IMPL1 and IMPL2 activity was determined at 22°C (reaction conditions for IMPL1 and IMPL2 as described in Methods). The kinetic parameters were obtained from the initial velocity as described in Methods. The concentration of substrates was varied from 0 to 1 mM*.

For IMPL1, various substrates were tested (Table 1). D-Ins 1-P can be derived from Ins(1,4,5)P_3_ second messenger breakdown, in contrast to D-Ins 3-P, which is an intermediate in *de novo* inositol synthesis. Interestingly, D-Gal 1-P is hydrolyzed by IMPL1 (Table 1), which is similar to the action of the human IMP which hydrolyzes D-Gal 1-P as effectively as D-Ins 1-P (Parthasarathy et al., 1997). β-Glycerophosphate can also be hydrolyzed (39.7% of the D-Ins 1-P rate). Under these reaction conditions, D-Ins 3-P, D-Ins 2-P, L-Gal 1-P, adenosine 2′-monophosphate and D-Glc 1-P are hydrolyzed at a lower rate. In addition, glycerol 3-phosphate, D-glucose 6-P, D-mannitol 1-P, D-sorbitol 1-P, D-fructose 1-P and Fru 1,6-bisP, NADP, NADPH and PAP are not hydrolyzed at all by IMPL1. IMPL1 is also not able to hydrolyze the poly-phosphorylated inositol compounds (Table 1). Together, these data suggest that IMPL1 has distinct substrate specificity as compared to either IMPL2 or IMP, and might be involved in hydrolysis of D-Ins 1-P and/or D-Gal 1-P.

Catalytic properties of enzymes are important factors in determining substrate specificity of an enzyme. In reaction conditions of pH 9, 3 mM MgCl_2_, and 452 ng of IMPL1 recombinant enzyme, the *K_m_* for D-Ins 1-P was 180 ± 3 μM (Figure 1) and that for D-Gal 1-P was approximated to be 450 ± 60 μM. Substrate inhibition occurred at concentrations greater than 1 mM of D-Ins 1-P. The *k*_cat_-value for IMPL1 with D-Ins 1-P is 0.6 ± 0.1 s^−1^ and 2.4 ± 1.3 s^−1^ with D-Gal 1-P. Further, the ratio of *k*_cat_ to *K_m_* provides a perspective on the catalytic efficiency of an enzyme with a specific substrate, and the calculated *k*_cat_/*K_m_* with D-Ins 1-P is 3.3 ± 0.1 × 10^3^ M^−1^ s^−1^, and 5.3 ± 0.5 × 10^3^ M^−1^ s^−1^ with D-Gal 1-P (Table 2).

**Figure 2:**
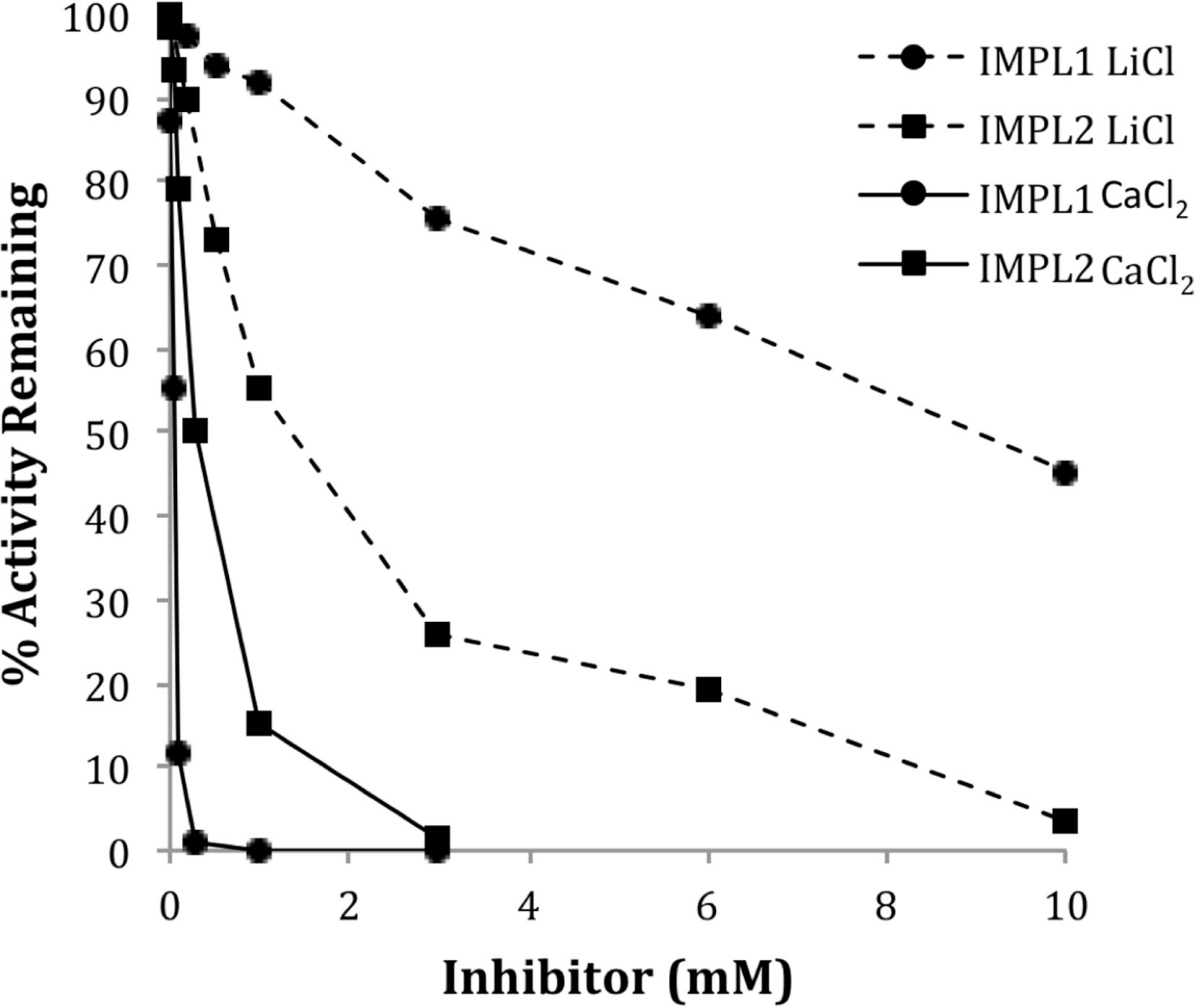
**Inhibition of IMPL1 and IMPL2 Activity by either LiCl or CaCl_2_**. IMPL1 activity was assayed with D-Ins 1-P (circles) and IMPL2 was assayed with histidinol 1-P (squares) in the presence of the indicated concentrations of CaCl_2_ (solid lines) or LiCl (dashed lines).

Lithium and calcium (Ca^2+^) ions have an inhibitory effect on other IMPs (Leech et al., 1993; Parthasarathy et al., 1997; Torabinejad et al., 2009). IMPL1 and IML2 are both inhibited by Li^+^ or Ca^2+^ addition (Figure 2), albeit this inhibition occurs at a high level of substrate such that these ions may be inhibiting the enzymes by complexing with substrate and displacing Mg^2+^. Interestingly, these data suggest that Li^+^ contamination of soil could impact IMPL2 function and histidine biosynthesis in plants. Indeed, several incidents of lithium toxicity in field-grown citrus with lithium concentrations of 0.06–0.1 ppm in the irrigation water has been reported in the state of California (Bradford, 1963).

### IMP and IMPL gene expression is temporally and spatially regulated

**Figure 3:**
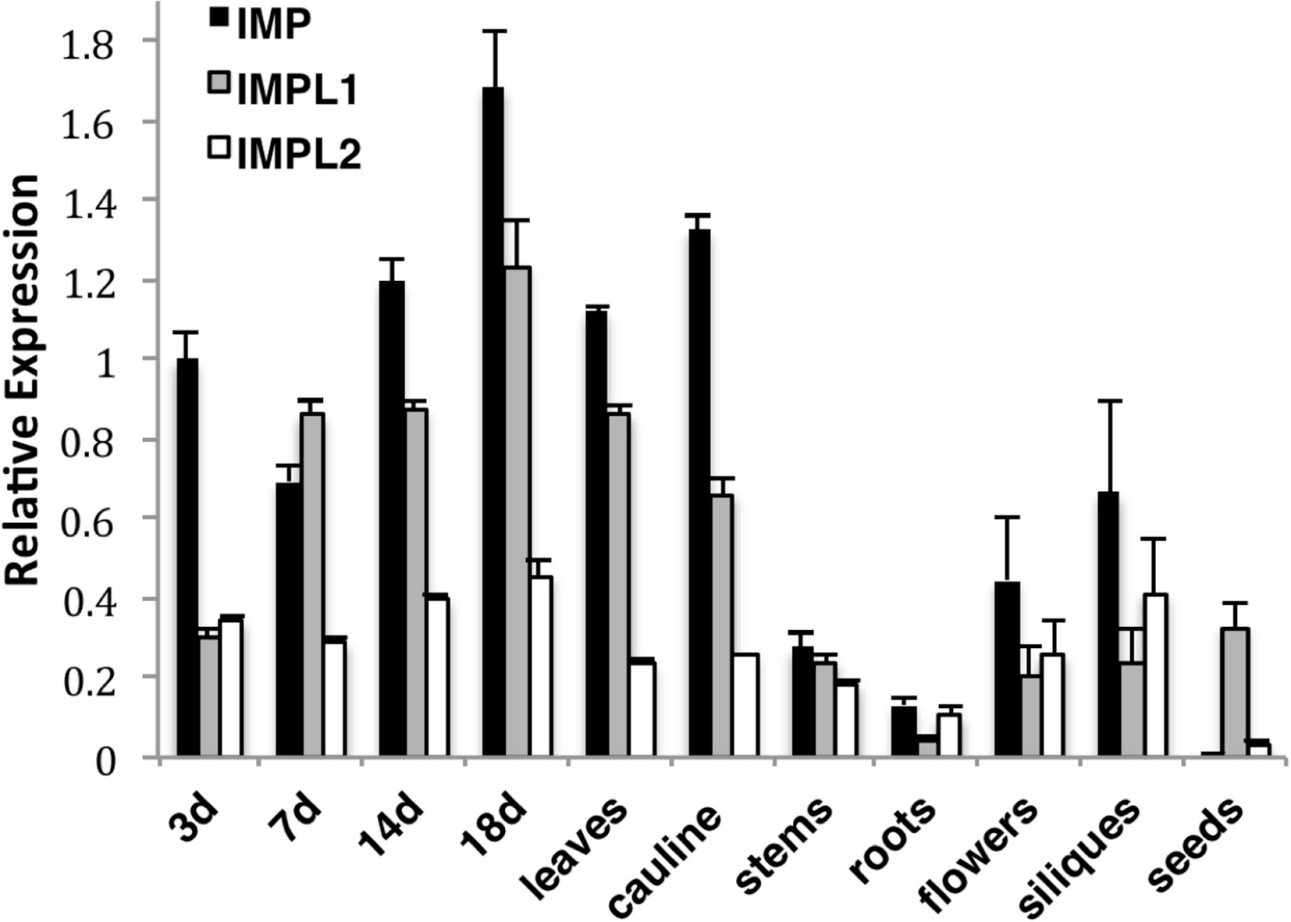
**Relative Expression of IMP and IMPL Genes as Determined by Real-time PCR**. IMP, IMPL1, IMPL2 gene expression was measured in 3,7,14-d-old wild type seedlings grown on 0.5× MS plus 1% sucrose-soaked filter paper under 16-h-light conditions, soil-grown 18-d-old whole plants (18d), young rosette leaves (leaves), roots, cauline leaves (cauline), stems, flowers, and immature siliques from 35-d-old plants and seeds imbibed in water for 3 d at 4°C. Real-time PCR amplification curves of genes of interest were compared with PEX4 (peroxisomal ubiquitin4) amplification to generate relative expression levels. PEX4 was used as an endogenous control because it is expressed constitutively at all stages of development. Means of triplicate reactions of three biological replicates ± SE are presented.

To determine whether transcription of IMP and IMPL genesis differentially regulated, we performed quantitative PCR to compare relative expression of IMP, IMPL1, and IMPL2 in various tissues (Figure 3). We found that IMP is expressed in all tested tissues except seeds and levels are high in seedlings, leaves, and cauline leaves during early development. IMPL1 has a similar expression pattern as IMP, however it is expressed at slightly lower levels, and it is the only IMP gene abundantly expressed in seeds. IMPL2 expression is overall lower as compared to IMP and IMPL1, and IMPL2 appears to be expressed constitutively in all tissues except seeds. The results are similar to those reported from microarray data provided by Genevestigator database (Zimmermann et al., 2004) (Supplemental Figure 2).

**Figure 4:**
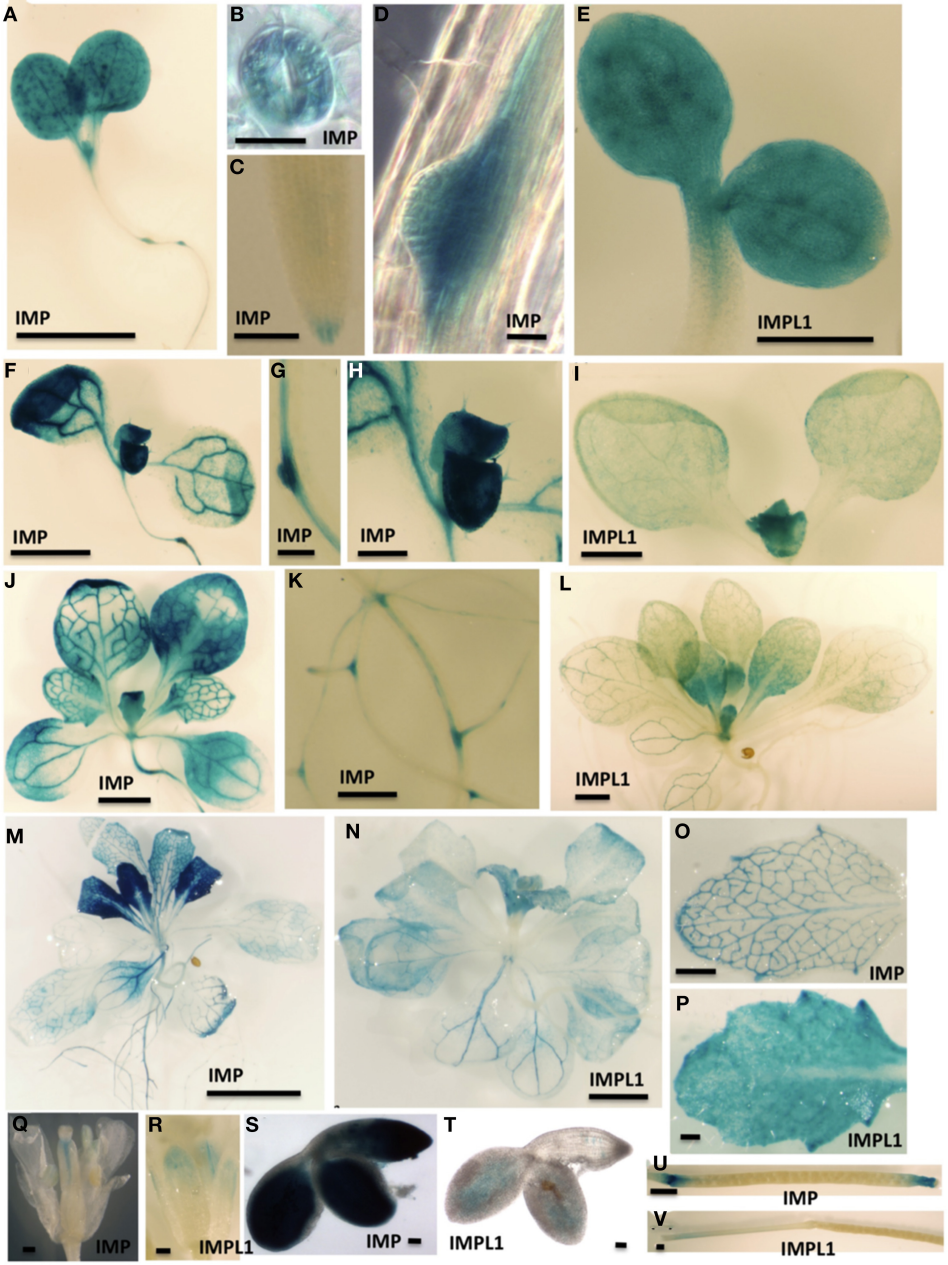
**Spatial Expression Patterns of IMP and IMPL1 Genes**. The promoters from IMP and IMPL1 were used to drive GUS expression in transgenic plants. **(A–E)** Three-day-old seedlings grown on 0.5× MS plus 1% sucrose. Bars = 1 mm in **(A)**, 20 μm in **(B,D)**, 200 μm in **(C)**, and 500 μm in **(E)**. **(F–I)** Seven-day-old seedlings grown on 0.5× MS plus 1% sucrose. Bars = 1.3 mm in **(F)**, 200 μm in **(G)**, 377 μm in **(H)**, and 1 mm in **(I)**. **(J–L)** Fourteen-day-old seedling grown on 0.5× MS plus 1% sucrose. Bars = 2 mm in **(J,L)**, and 500 μm in **(K)**. **(M,N)** Nineteen-day-old seedling grown on 0.5× MS plus 1% sucrose. Bars = 5 mm in **(M)** and 2 mm in **(N)**. **(O–V)** Organs from soil-grown plants. **(O,P)** Leaves. Bars = 50 μm in **(O)**, and 200 μm in **(P)**. **(Q,R)** Flowers. Bars = 500 μm.

To investigate the spatial pattern of regulation of the IMP and IMPL genes, we sought to generate transgenic plants expressing IMP, IMPL1, and IMPL2 promoters fused to the *uidA* gene, which encodes β-glucuronidase (GUS). Several independent transgenic lines for ProIMP-*uidA* and ProIMPL1-*uidA* constructs were analyzed and consistent patterns were detected in ProIMP-*uidA* 3-d-old seedlings, β-glucuronidase (GUS) activity was noted in the entire cotyledon, within the upper hypocotyl, leaf primordia, lateral root primordia, primary root tips, and guard cells (Figures 4A–D). ProIMPL1-*uidA* shows a similar pattern in 3-d-old seedlings, however IMPL1 is not expressed in root tissue (Figure 4E and not shown). In 7-d-old seedlings, IMP expression is prevalent in the vascular tissue in cotyledons, roots, and leaves, and trichomes (Figures 4F–H). At 7-d, IMPL1 expression is weakly maintained in the cotyledons but expression in leaf primordia is stronger (Figure 4I). In 14-d-old plants, IMP expression is similar to 7-d seedlings with vascular expression in most leaves and within roots (Figures 4J,K). At 14-d, IMPL1 expression is highest in young sink leaves, and is restricted to vascular tissue within older, source leaves (Figure 4L). In 19-d-old plants, IMP expression is observed in all cells of young, sink leaves and becomes restricted to vascular tissue within older, source leaves (Figure 4M). The expression of IMP in 19-d-old roots remains the same as in the earlier stages of development (Figure 4K). At 19-d, the IMPL1 expression pattern is similar to that of IMP, however expression is restricted to the shoot (Figure 4N and not shown). Leaves from soil-grown plants indicate that IMP expression is restricted to the vascular tissue and IMPL1 is expressed throughout the leaf (Figures 4O,P). In flowers, IMP is expressed in the pistil while IMPL1 expression is present in vascular tissue in the sepals (Figures 4Q,R). Both genes are expressed in the mature embryo, however, once again, IMPL1 is restricted to the shoot portion of the embryo (Figures 4S,T). Within siliques, IMP is expressed in the tips and abscission zones of immature siliques (Figure 4U), while IMPL1 is restricted to the stem of the immature silique (Figure 4V). Together, these data indicate that the IMP and IMPL1 genes are developmentally and spatially regulated in a similar fashion. One exception to this is that IMPL1 expression is restricted to shoot tissues, while IMP is expressed in both shoots and roots.

We have analyzed multiple transgenic plant lines containing four different IMPL2 promoter:*uidA* constructs, and have been unsuccessful in obtaining lines that show expression in any tissue. For this work we examined 1628 bp, 1085 bp or 461 bp upstream of the start site of transcription and the entire genomic sequence. We therefore conclude that it is likely that sequences outside of the promoter are necessary for dictating IMPL2 expression.

### The IMP protein is located in the cytosol and IMPL proteins are located in the chloroplast

**Figure 5:**
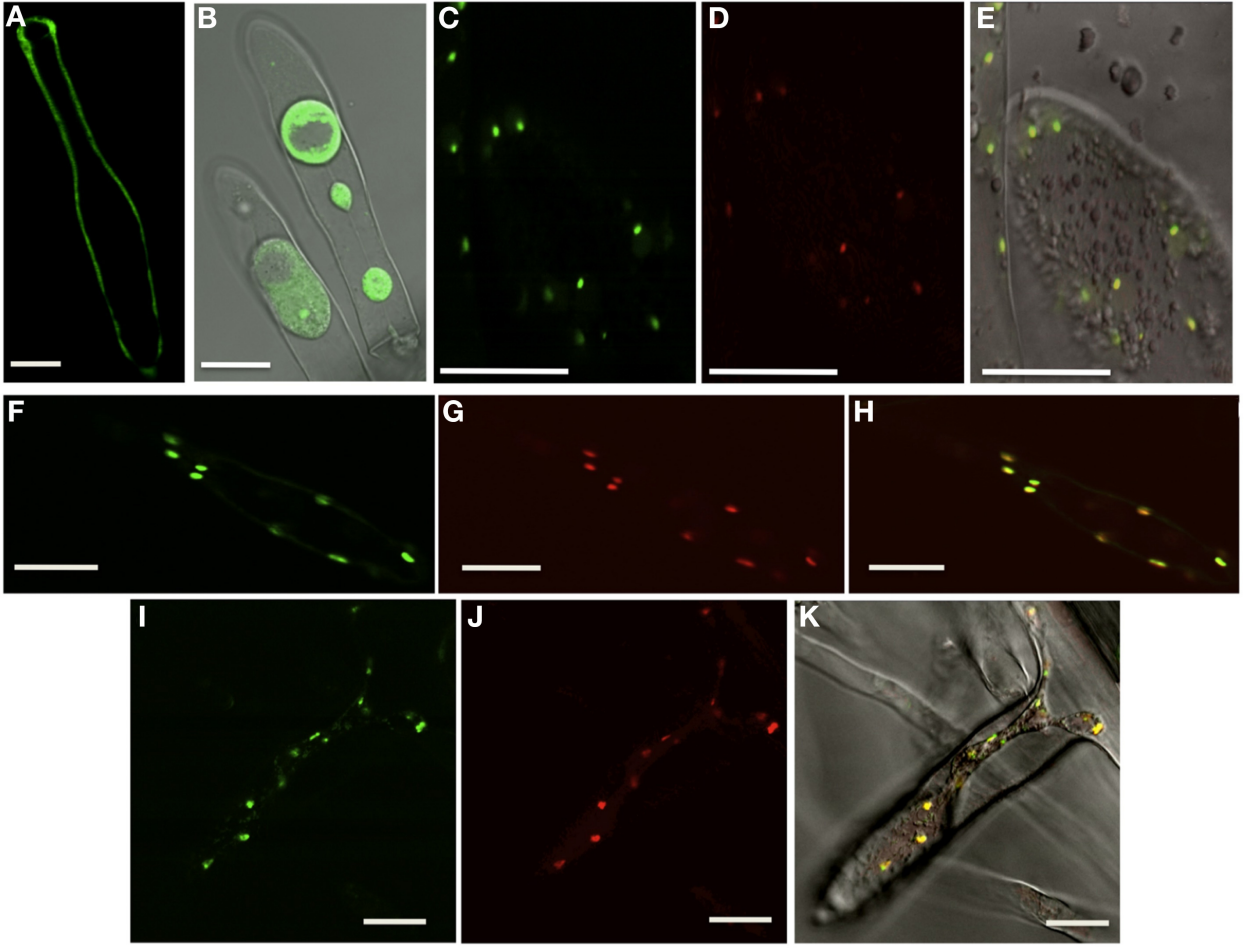
**Subcellular Location of IMP, IMPL1, and IMPL2 Proteins**. Single optical sections of transgenic plants expressing IMP:GFP **(A,B)**, IMPL1:GFP **(C)**, IMPL2:RFP **(D)**, overlay of IMPL1:GFP/IMPL2:RFP **(E)**, IMPL2:GFP **(F)**, Plastid mcherry **(G)**, overlay of IMPL2:GFP/plastid-mcherry **(H)**, IMPL1:GFP **(I)**, Plastid mcherry **(J)**, overlay of IMPL1:GFP/plastid-mcherry **(K)**. All images were taken of root hairs with differential interference contrast (DIC) overlay of plasmolyzed cells **(B)**, DIC overlay of co-localizations **(E,K)**. Bars = 20 μm.

Both IMPL1 and IMPL2 have been localized to the chloroplast in transient expression assays and in proteomics analysis of chloroplasts (Sun et al., 2009; Petersen et al., 2010). To investigate the subcellular location of IMP and IMPL proteins in multiple tissues, we constructed transgenic plants expressing IMP:GFP, IMPL1:GFP or IMPL2:GFP under the control of the 35S cauliflower mosaic virus (CaMV) promoter (Figure 5). We analyzed homozygous progeny from two independent lines for each construct with confocal microscopy and found similar patterns. Western blot analysis confirmed that intact fusion proteins accumulate (Supplemental Figure 3). For IMP:GFP, GFP fluorescence was predominantly associated with the cytoplasm in 3-d-old light-grown seedling shoots and roots (roots are shown in Figure 5A). Plasmolysis with 800 mM NaCl confirmed the cytoplasmic location (Figure 5B).

As expected, we found that IMPL1:GFP and IMPL2:GFP localized to small organelles in root and shoot tissues (Figures 5C,F). In addition, co-localization of IMPL1:GFP and IMPL2:RFP fusion proteins from plants expressing both indicate that both are present in the same compartment (Figures 5C–E). To confirm this, we transformed IMPL2:GFP and IMPL1:GFP transgenic plants with a plastid-mcherry marker containing the signal peptide of the pea Rubisco small subunit (Nelson et al., 2007). The data demonstrate that both IMPL1 and IMPL2 proteins are directed to plastids (Figures 5E–K).

IMPL1 and IMPL2 proteins have N-terminal extensions of 77 amino acids that are predicted to function as transit peptides and are not present in homologous IMP proteins. To determine whether these predicted transit peptides are sufficient for organelle targeting, these N-terminal extensions were fused to GFP. The resulting constructs, Pro35S:NterIMPL1:GFP and Pro35S:NterIMPL2:GFP were stably transformed and the putative IMPL2 signal peptide directed plastid expression of GFP similar to that seen with IMPL2:GFP localization (Supplemental Figure 4). The 77 amino acid putative transit peptide from IMPL1 also was sufficient for localization to plastids, however the intensity of expression was significantly reduced (Supplemental Figure 4). From these data, we conclude that the N-terminal 77 amino acids on both IMPL1 and IMPL2 are sufficient for localization to plastids.

### Characterization of *impl2* mutants

**Figure 6:**
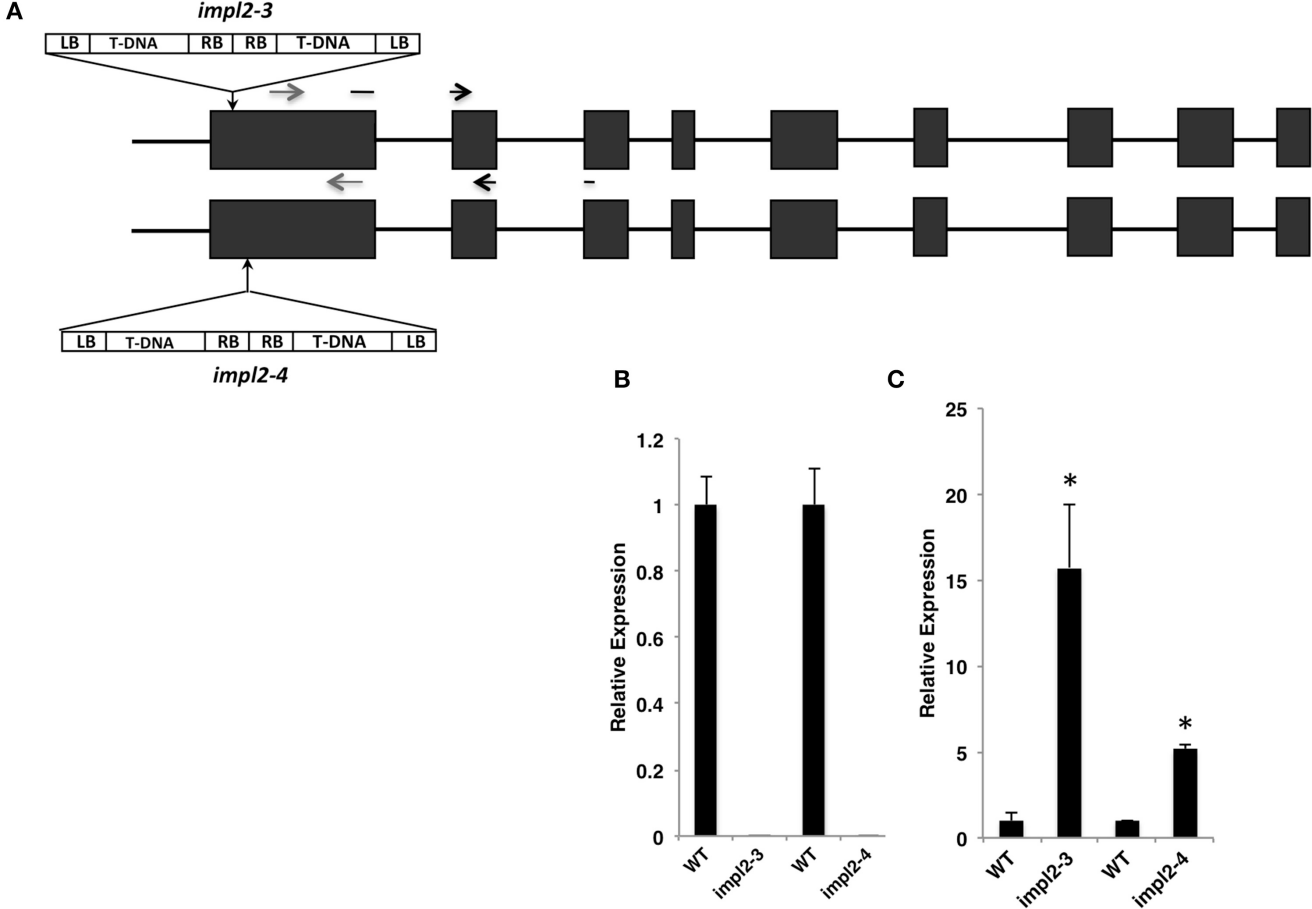
**T-DNA Insertions and Mutant Gene Expression. (A)** Schematic of T-DNA insertion sites in the *impl2-3* and *impl2-4* mutants. Exons are shown as dark-gray boxes; the gray arrows indicate primers that are used in **(B)**; black arrows indicate primers that are used in **(C)**. **(B, C)** Expression levels of IMPL2 gene in 21-d-old wild-type and mutant plants. Real-time PCR amplification (see Methods) was compared with PEX4 amplification to generate relative expression levels. Means of triplicate biological reactions ± SE are represented. Asterisks indicate significant difference from the wild-type (*p* < 0.01) in a Student's *t*-test.

To determine how the IMPL2 gene impacts histidine synthesis and plant growth and development, T-DNA insertion mutants were obtained from the SALK T-DNA insertion collection (Alonso et al., 2003). Seeds for *impl2-3* (SAIL_35_A08) and *impl2-4* (SAIL_146_E09) were obtained, and homozygous mutants were verified by diagnostic PCR screening and DNA sequencing, as described in the experimental procedures. The *impl2-3* mutant contains two tandem T-DNA insertions occurring 24 nucleotides from the start of translation (Figure 6), and is the same line identified previously as an embryo-lethal (Petersen et al., 2010). The *impl2-4* mutant contains two tandem T-DNA insertions 66 nucleotides from the start of translation (Figure 6). Lack of full-length IMPL2 gene expression was verified in the mutants by qPCR (Figure 6). Interestingly, we detected an increased presence of truncated transcript in both mutants using primers downstream of exon one (Figure 6). Thus, there is a possibility that a functional or non-functional IMPL2 protein accumulates in the cytosol of these mutants.

### The *impl2* mutants are altered in growth and development

Previous examination of *impl2-3* mutants indicated homozygosity leads to embryo lethality, and histidine application to heterozygous plants can rescue seed development (Petersen et al., 2010). However, we were able to obtain homozygous progeny of both *impl2-3* and *impl2-4* that produce viable seeds. We analyzed two other T-DNA insertion mutant lines, *impl2-1* and *impl2-2*, but were not able to recover homozygous progeny, strongly suggesting embryo lethality within these lines. Analysis of 30 siliques from wild-type and heterozygous *impl2-1* mutants revealed that approximately 25% of the *impl2-1* seeds were dark and shriveled, while less than 1% of wildtype seed had this appearance, suggesting embryo lethality of homozygous *impl2-1* seed.

**Figure 7:**
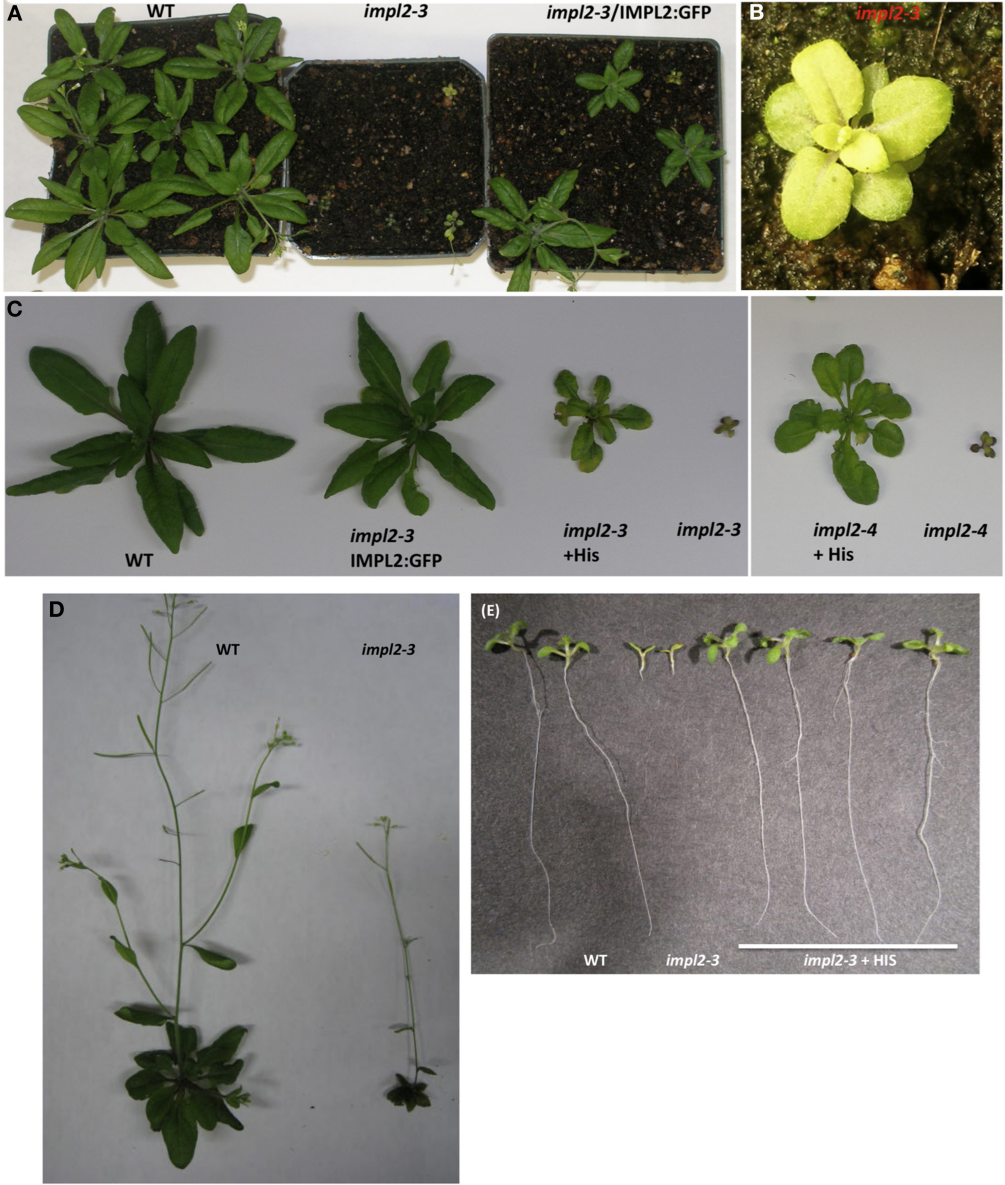
**Histidine or IMPL2-GFP Gene Complement the Stunted Stature of *impl2* Mutants. (A)** Segregation of progeny from heterozygous *impl2-3* plants containing 35S promoter-IMPL2-GFP. **(B)** Image of *impl2-3* rosette exhibiting small, pale green leaves **(C)** Soil-grown *impl2-3*, *impl2-4*, wild-type (CS60000) and complemented plants. Mutant plants were watered with 1 mM histidine. **(D)** Soil-grown wild-type and *impl2-3* plants. **(E)** Photos of 9-d-old wild-type and *impl2-3* seedlings grown on agar plates for root length studies. Root phenotype of *impl2-3* is complemented by the addition of 0.04 mM histidine.

The *impl2-3* and *impl2-4* mutant plants are severely compromised in growth and exhibit several main phenotypes, which are quantified in Table 3. These phenotypes include smaller size, reduced inflorescences and seed production (Figure 7). To ensure that these phenotypes result from an IMPL2 loss-of-function, we complemented *impl2-3* with a 35Spromoter: IMPL2:GFP transgene. These complemented plants (*impl2-3/*IMPL2:GFP) exhibited wild-type or near wild-type phenotypes in several different assays (Figures 7A,B). This, along with the finding of two separate mutant alleles (*impl2-3* and *impl2-4*), strongly supports alteration in IMPL2 function as the primary cause for our observed growth phenotypes.

**Table 3:** **Overview of the *impl2-3* and *impl2-4* mutant phenotype**.

	**Wild type**	***impl2-3*^a^**	***impl2-3* + histidine**	***impl2-3* IMPL2:GFP**	***impl2-4*^b^**
Rosette diameter (cm)	4.87 ± 0.2	1.10 ± 0.1^*^	4.14 ± 0.2	3.69 ± 0.2	1.6 ± 0.1^*^
Number of rosette leaves per plant	18.3 ± 0.7	9.4 ± 0.5^*^	17.1 ± 1.2	17.3 ± 0.9	12.1 ± 0.6^*^
Average rosette leaf surface (cm^2^)	2.03 ± 0.1	0.20 ± 0.02^*^	2.03 ± 0.1	1.57 ± 0.1	0.28 ± 0.02^*^
Number of inflorescence stems per plant	6.6 ± 0.5	1.9 ± 0.3^*^	7.2 ± 0.8	4.6 ± 0.6	1.8 ± 0.2^*^
Weight of seeds per 6 plants (mg)	226 ± 9	51.7 ± 2^*^	247 ± 19	190 ± 3	65.3 ± 4.1^*^

*Rosettes and stems were measured 9 weeks after germination. Seeds were harvested, and weighed after they were dried. Data represents the means ± SE; n = 20 for rosettes and stems; n = 4 for seeds*.

a,b Asterisks indicate values found to be significantly (Student's t-test) different from the wild type:

* **p* < 0.005*.

**Figure 8:**
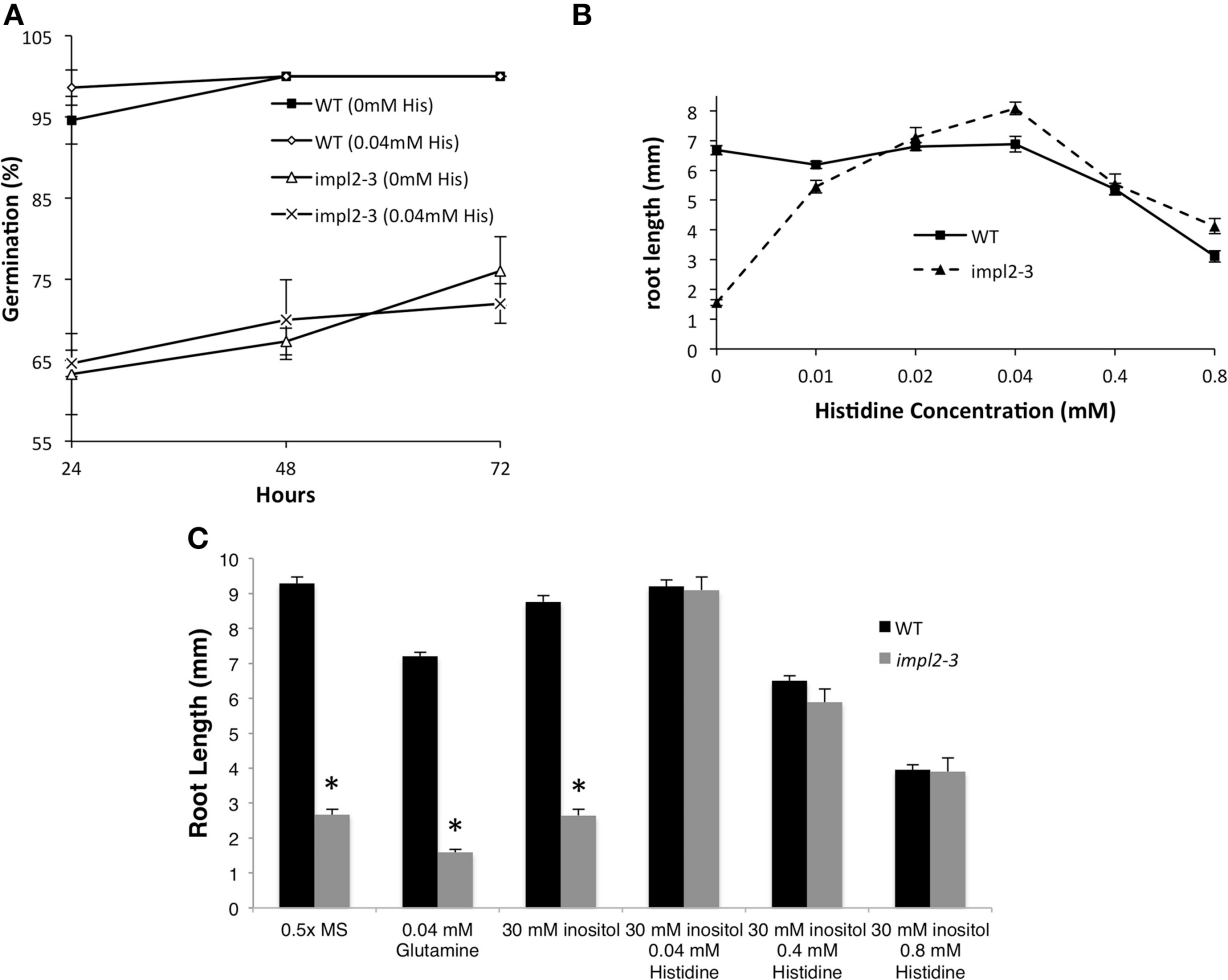
**Physiological Responses of *impl2-3* Mutants to Exogenous Histidine and Inositol**. **(A)** Effects of histidine on germination of the wild-type and *impl2-3* mutants grown on agar plates. **(B)** Dose Response of 4-d-old wild-type and *impl2-3* mutant seedlings grown for root length studies on agar plates with the indicated histidine concentrations. **(C)** Effects of glutamine, inositol and histidine on root length of wild-type and *impl2-3* mutants grown on agar plates. Presented are means ± SE of three experiments of *n* = 50 (germination) and three experiments of *n* = 30 (root length). Asterisks indicate values found to be significantly (Student's *t*-test) different from the wild type: ^*^p < 0.005.

Although both *impl2* mutant lines show very similar phenotypes throughout development, *impl2-3* has been the focus for our experiments. We analyzed the germination rate of mutant seeds and noted that only 75% of *impl2-3* seeds germinate, while 97.5% of WT seeds germinate (Figure 8A). After germination of *impl2-3* mutant seeds, we noted significant delay in seedling development as compared to wild-type seedlings, which continues throughout development. Homozygous *impl2* mutants are overall smaller than wild-type plants (Figure 7 and Table 3); *impl2* mutant roots do not grow well (Figures 8B,C), and most seedlings do not produce true leaves and die after a few days. The seedlings that develop beyond this stage are able to produce true leaves, however the leaves are a pale green color (Figure 7B), and roots remain stunted. Mutant cotyledons and leaves were observed by microscopy; the overall structure of chloroplasts appeared similar to those in wild-type plants (data not shown). The *impl2* plants that survive to maturity produce very few siliques, and some viable seeds (Table 3).

To test whether histidine deficiency is responsible for the altered development of *impl2* mutants, we watered *impl2* mutants and wild-type plants with 1 mM histidine, with a control amino acid, glutamine (Figures 7, 8). The results show that continuous watering with 1 mM histidine (Figures 7, 8) but not 1 mM glutamine (data not shown), alleviates much of the severe growth reduction in *impl2* mutants. To test whether histidine application could rescue *impl2* seed germination and seedling defects, we produced age-matched seed populations that had been harvested from plants grown at the same time. Control and mutant age-matched seeds were plated on Murashige and Skoog (MS) medium in the presence of various concentrations of histidine, glutamine and/or inositol. Our results indicate that *impl2-3* mutants germinate at the same rate in the presence or absence of histidine (Figure 8A). However, root growth of *impl2* mutants is restored to wild-type levels in the presence of histidine, while neither glutamine nor inositol improves root growth of these mutant plants (Figure 8C). The optimal range for chemical complementation with exogenous histidine is 0.02–0.04 mM, and larger concentrations such as 0.4 or 0.8 mM of histidine have an inhibitory effect on root growth of both *impl2* mutant and wild-type plants grown on agar plates (Figure 8B). The fact that exogenous inositol added to medium was not able to alleviate the stunted root phenotype of *impl2* mutants (Figure 8), suggests that IMPL2 is not involved in inositol synthesis or inositol phosphate metabolism.

### IMPL2 impacts histidine synthesis

To determine if a loss in IMPL2 function impacts histidine biosynthesis, we used LC-MS-MS to quantify histidine levels in wild-type and *impl2-3* mutants (Table 4). Amino acids were extracted using 1:1 chloroform: 10 mM HCl (v/v) and norvaline was used as internal standard. Standard curves and interpretation of MS data are described in the Supplemental Methods.

**Table 4:** **Histidine and histidinol levels at different developmental stages**.

**Tissue**	**WT (μmoles mg DW^−**1**^)**	***impl2-3*^a^ (μmoles mg DW^−**1**^)**	***impl2-3* IMPL2:GFP (μ moles mg DW^−**1**^)**	**IMPL2:GFP (μ moles mg DW^−**1**^)**
histidine 7-d	0.15 ± 0.01	0.27 ± 0.01	0.28 ± 0.01	0.15 ± 0.01
histidine 18-d	0.25 ± 0.01	0.38 ± 0.01	0.37 ± 0.01	0.35 ± 0.02
histidine 31-d	0.35 ± 0.01	0.35 ± 0.01	0.36 ± 0.01	0.35 ± 0.01
histidinol 7-d	0.0092 ± 0.0001	0.33 ± 0.01^*^	0.0010 ± 0.0001	0.0009 ± 0.0001
histidinol 18-d	0.0010 ± 0.0002	0.48 ± 0.01^*^	0.0008 ± 0.0001	0.0008 ± 0.0001
histidinol 31-d	0.0007 ± 0.0001	0.34 ± 0.02^*^	0.0009 ± 0.0002	0.0009 ± 0.0001
histidine +	1.87 ± 0.03	2.10 ± 0.02	NM	NM
histidinol +	0.54 ± 0.02	1.03 ± 0.02	NM	NM

*Seedlings and plants were grown on 0.5× MS, pH 5.8, and 1% sucrose. Seedlings of 7-d-old, 18-d-old or whole rosette and roots of 31-d-old wild-type, impl2-3, impl2-3 IMPL2-GFP, and IMPL2-GFP plants were harvested, and histidine and histidinol levels were quantified with LC-MS-MS as described in Methods. ^+^histidine and histidinol levels were measured in 7-d-old seedlings that were grown on 0.8 mM histidinol (NM = not measured). Data represents the means ± SE; n = 3*.

a Asterisks indicate values found to be significantly (Student's t-test) different from the wild type:

* **p* < 0.005*.

In 7-d-old seedlings, histidine levels are slightly increased in *impl2-3* mutants as compared to wild-type, and the levels are not rescued to wild-type levels in the complemented plants (Table 4). Histidine levels remain elevated in 18-d mutants as compared to wildtype plants. Interestingly, later in development (31 days), whole plants from *impl2-3* mutants show levels of free histidine equal to that found in wild-type, indicating that the amount of histidine is not altered in the *impl2* mutants at this time in development.

We also sought to measure histidinol 1-P, the substrate of IMPL2, and histidinol, the product of IMPL2 catalysis of histidinol 1-P. After numerous attempts, we found we could not detect histidinol 1-P in any plant extract. In contrast, although levels of histidinol were low in wild-type plants, we could reproducibly quantify this compound (Table 4). Since a common issue with metabolite extraction of phosphorylated compounds is hydrolysis of phosphates during sample extraction and derivatization, we tested whether the histidinol measured in our assays could result from the breakdown of histidinol 1-P during sample preparation. We added 100 μmoles of purified histidinol 1-P to wild-type tissue during the extraction procedure along with the addition of internal standard, norvaline, and found that in wild-type extracts where no histidinol 1-P was added, histidinol levels are barely detectable (0.001 ± 0.002 μmoles mg dried weight^−1^). Conversely, in the wild-type extract with added 100 μmoles of histidinol 1-P, histidinol levels are increased by 100-fold to a concentration of 0.1 ± 0.01 μmoles mg dried weight^−1^ (Supplemental Figure 5). Our conclusion is that our histidinol peak from LC-MS-MS analyses of plant extracts likely gives us information on the histidinol plus histidinol 1-P concentration in mutants and wild-type plants.

Using this methodology, we measured the histidinol plus histidinol 1-P in *impl2* mutants and wild-type plants. We found that *impl2-3* 7-d-old seedlings accumulated 0.33 ± 0.01 μmoles mg dried weight^−1^ as compared to the barely detectable wild-type levels of 0.0092 ± 0.0001 μmoles mg dried weight^−1^ (Table 4). This trend for higher levels was seen at 18-d and 31-d as well. This suggests that lack of histidinol 1-P hydrolysis in *impl2* mutants results in accumulation of precursors in the histidine pathway. Importantly, in IMPL2 complemented plants and IMPL2:GFP plants, histidinol plus histidinol 1-P levels at 7, 18, and 31 days are similar to those from wild-type plants (Table 4). Thus, the elevation of precursors in the histidine pathway correlates with the altered growth and development of *impl2* mutants.

**Figure 9:**
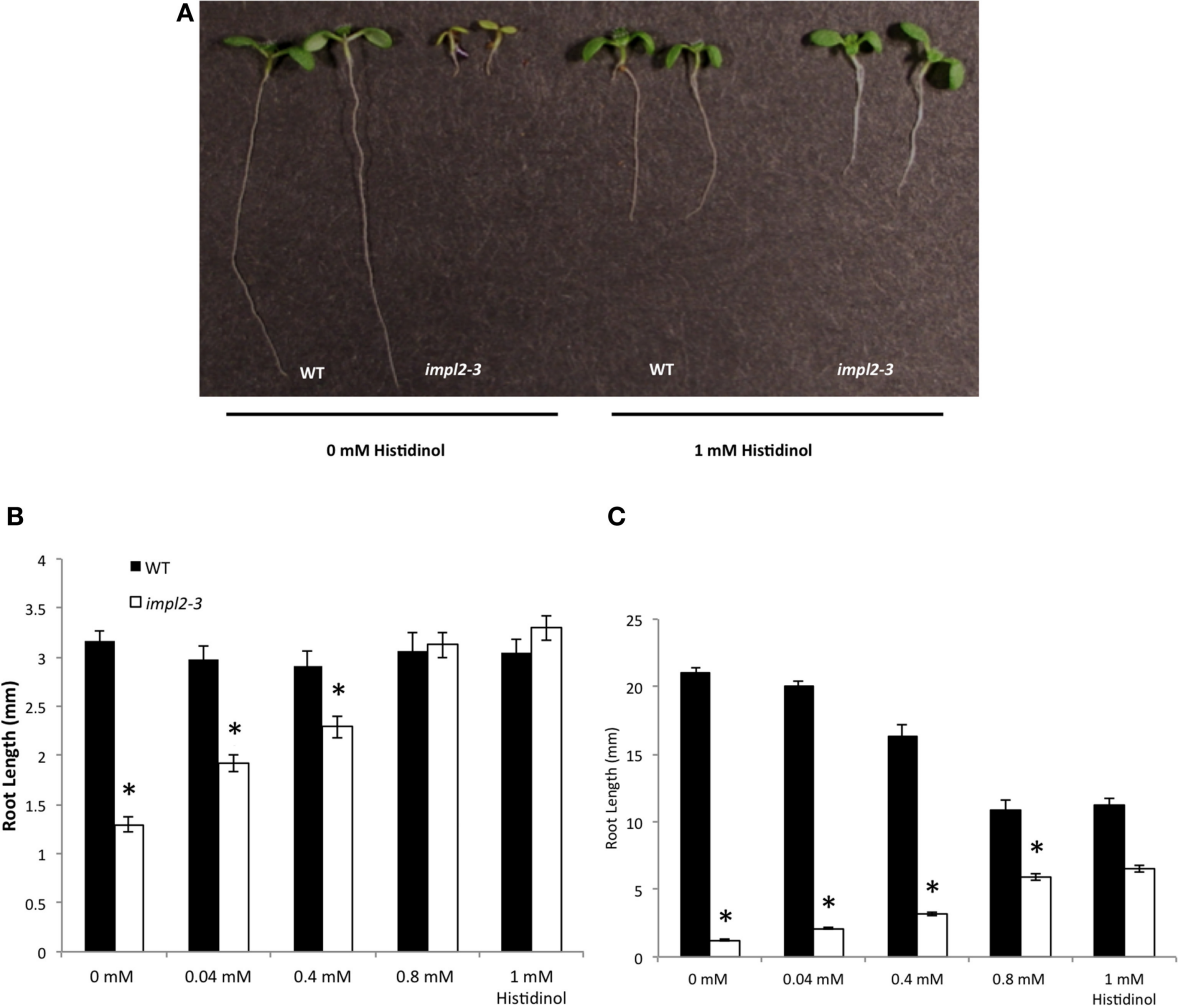
**Chemical Complementation of *impl2-3* Mutants with Exogenous Histidinol. (A)** Photos of 8-d-old wild-type and *impl2-3* seedlings grown on agar plates for root length studies. Root phenotype of *impl2-3* is complemented by the addition of 0.8–1 mM histidinol. **(B)** Dosage Response of 4-d-old **(C)** 8-d-old wild-type and *impl2-3* mutant seedlings grown for root length studies on agar plates with the indicated histidinol concentrations. Presented are means ± SE of *n* = 40, ^*^p < 0.05.

To test whether *impl2* mutants can be rescued by histidinol, we grew *impl2-3* and wild-type seeds in the presence of varying concentrations of histidinol (Figure 9). The root length phenotype of *impl2-3* seedlings was complemented by 0.8–1 mM of histidinol by day 4 and this amount was not toxic to the growth of wild-type seedlings. However, at 8 days the histidinol started to have an inhibitory effect on growth in both WT and *impl2-3* mutant plants. We conclude that exogenous histidinol can rescue the growth of *impl2-3* mutants, however accumulation of high levels of histidinol can exhibit an inhibition in growth further in development. Thus, our developmental analysis and histidine metabolite data analyses firmly establish that *impl2* mutants have alterations in the histidine biosynthetic pathway that lead to severe growth alterations, and underscore the importance of this pathway in plant growth and development.

### An IMPL2 loss-of-function does not impact myo-inositol levels

Given the bifunctionality of several of the characterized IMPs, we wanted to rule out the possibility that IMPL2 can impact inositol levels by *in vivo* hydrolysis of D-Ins 1-P or D-Ins 3-P. We quantified inositol and six other metabolites, including ascorbic acid, a downstream product that can result from inositol catabolism. No difference in inositol levels was observed in *impl2-3* 7-d-old seedlings as compared to wild-type seedlings, however, fructose, ascorbic acid, glycerate, and xylose levels were altered in these mutants (Supplemental Figure 6). Given the substrate specificity of recombinant IMPL2-GST and the lack of inositol alterations in *impl2* mutants, we conclude that IMPL2 plays little to no role in inositol synthesis or recycling in the plant cell. We also examined IMPL1 overexpressing plants (Supplemental Figure 6). We found that inositol levels were not altered in these plants. However, as was true for the *impl2-3* mutants we found that overexpression of IMPL1:GFP resulted a small elevation of ascorbic acid (Supplemental Figure 6).

## Discussion

IMP enzymes have been a focus of study in plants since the pioneering work of Frank Loewus in the 1960s (Loewus and Kelly, 1962; Loewus et al., 1962; Loewus, 1964, 1965, 1969). Given that the canonical IMP in plants is bifunctional, hydrolyzing both inositol phosphates involved in *de novo* inositol synthesis and inositol signaling, and L-Gal 1-P, a precursor to ascorbic acid (Torabinejad et al., 2009), we wanted to address the functionality of the IMPL enzymes. We were guided by work from Petersen et al. that IMPL2, but not IMPL1, is sufficient to rescue the histidine auxotrophy of a *Streptomyces coelicolor* hisN mutant, which is defective in His 1-P phosphatase activity (Petersen et al., 2010). Our comparison of IMPL1 and IMPL2 recombinant protein activity using a variety of substrates, along with genetic characterization of metabolite levels in viable *impl2* mutants, solidifies the role of IMPL1 in inositol and/or galactose phosphate metabolism, and IMPL2 in the histidine synthesis pathway.

### IMPL2 is not a moonlighting enzyme

The fact that IMPL2 can rescue histidine auxotrophy of a *Streptomyces coelicolor* hisN mutant (Petersen et al., 2010) suggested IMPL2 either functioned in both inositol and histidine synthesis (i.e., a moonlighting activity), or had diverged in its substrate specificity. Our biochemical examination shows that IMPL2 has specificity for His 1-P, and our genetic and metabolite analyses of viable *impl2* mutants shows the importance of this reaction in the histidine synthetic pathway, with no apparent role in the inositol metabolic pathway.

### Biochemical evidence for histidinol 1-phosphate phosphatase activity

Key to our analysis of IMPL2 activity was the synthesis of the His 1-P substrate (provided by Robert White), which is not available commercially, and limits the ability of investigators to examine catalysis by these enzymes. We found that recombinant AtIMPL2 has a *K_m_* value slightly higher than other monofunctional His 1-P phosphatases characterized previously. The catalytic efficiency we delineated for AtIMPL2 is lower than those from unicellular organisms (Millay and Houston, 1973; Lee et al., 2008). In contrast, the AtIMPL2 *K_m_* value of 180 μ M is slightly different than the only other reported value from a partially purified plant His 1-P phosphatase activity (from wheat) estimated to be 0.4 mM (Wiater et al., 1971). The lack of hydrolysis of inositol phosphates or related molecules by IMPL2 clearly allows us to make a definitive statement that the IMPL2 is indeed the last missing enzyme in the plant histidine pathway (Petersen et al., 2010), and it does not play a role in inositol metabolism or signaling.

### The impact of IMPL2 on histidine synthesis and plant growth

The most common histidine-starvation phenotype in plants is embryo-lethal at the pre-globular stage (Muralla et al., 2007). In our search for a genetic loss of function mutant in IMPL2, we identified two embryo-lethals and two other viable, homozygous mutants, named *impl2-3* and *impl2-4*. Both mutant lines are greatly altered in growth and development, produce few seeds and can be rescued by exogenous histidine application. The *impl2-3* mutant has been previously reported to be embryo-lethal which can be rescued by exogenous histidine application. It is not obvious why we have been able to grow this same mutant and obtain progeny without histidine application, but one possible explanation is a difference in our growth conditions that may facilitate His 1-P breakdown in the mutants.

We complemented the *impl2-3* mutant with a 35S:IMPL2:GFP construct, which rescued the growth and production of histidine pathway precursors. It is interesting to note that our metabolite analyses indicated that *impl2-3* mutants, complemented mutants and IMPL2 overexpressors all had concomitant small changes in fructose, ascorbate, and xylose. We feel these changes are most likely resulting from our use of the 35S promoter, which clearly drives expression of IMPL2 to complement the growth of *impl2* mutants, but may not recapitulate the native pattern of IMPL2 expression. Thus, these metabolite differences may be linked to the decrease or relative increase in IMPL2 function in these plants.

### Function of IMPL1

Our biochemical experiments with recombinant IMPL1 indicate that it has no activity with His 1-P, as predicted from lack of genetic complementation in the Actinobacteria *histidine* auxotroph mutant (Petersen et al., 2010). From our kinetic studies, IMPL1 is most likely involved in hydrolyzing D-Ins 1-P and/or D-Gal 1-P. D-Ins 1-P is a breakdown product of D-Ins(1,4,5)P_3_ second messenger, while no role is yet known for D-Gal 1-P in plants, although the mammalian IMP is capable of hydrolyzing D-Gal -1-P (Parthasarathy et al., 1997). The IMPL1 substrate specificity is thus different from that of the plant IMP, which hydrolyzes D-Ins 1-P and D-Ins 3-P and L-Gal 1-P to similar degrees (Laing et al., 2004; Torabinejad et al., 2009). As we and others have provided evidence that IMPL1 is located in the chloroplast, this suggests that IMPL1 may be involved in recycling *myo*-inositol from InsP(1,4,5)P_3_ or another D-inositol phosphate within the chloroplast. It is interesting to note that IMP and IMPL1 are regulated similarly at the spatial level, except for the lack of IMPL1 expression in roots. Thus, for most above-ground tissues, IMP and IMPL1 could be functionally redundant with respect to breakdown of D-inositol phosphates. The role of signaling inositol phosphates in the chloroplast, is at present, unknown, however there is evidence for inositol synthesis within the chloroplast (Parker et al., 1987; Johnson and Wang, 1996). It is currently unknown whether chloroplasts synthesizes higher inositol phosphates or phosphatidylinositol phosphates that could be acted on by phospholipase C, resulting in Ins(1,4,5)P_3_. Interestingly, chloroplasts are capable of releasing Ca^2+^ (Johnson et al., 1995), and a chloroplast Ca^2+^ sensor has also been characterized (Weinl et al., 2008).

Without more definitive data, such as an IMPL1 genetic mutant, we cannot ascribe a clear function to IMPL1. It is of interest that no IMPL1 T-DNA insertion mutant lines have been identified, and our multiple attempts to produce IMPL1 RNAi lines have not been successful, suggesting that IMPL1 is an essential gene. An interesting clue to IMPL1 function comes from the *Chlamydomanas* IMPL1 homolog (called INM1), which is required for uniparental inheritance of chloroplast DNA in gametes, along with the key regulator for zygote development, *GSP1* (Nishimura et al., 2012). It has been shown that inactivation of the *Chlamydomonas* mating structure induces a rapid turnover of phosphatidylinositol(4,5)bisphosphate (Irvine et al., 1992; Musgrave et al., 1992), and it is speculated that this might drive Ins(1,4,5)P_3_ synthesis, stimulating the Ca^2+^/cAMP signal transduction system needed for successful mating and zygote development (Nishimura et al., 2012). If so, then IMPL1 (INM1) may be required for recycling of Ins(1,4,5)P_3_ in this system.

Given the similarity in sequence between IMP, IMPL1, and IMPL2, the difference in substrate specificity among these highly homologous enzymes is somewhat surprising.

Our work clearly delineates that the plant family of IMP and IMPL enzymes has evolved different substrate specificities, and that IMPL2 does not function in the inositol signaling pathway. In contrast, the IMPL1 enzyme appears to utilize similar substrates as the IMP enzyme, and the role of this chloroplast-localized IMPL1 enzyme awaits further investigation that could be greatly facilitated by a genetic mutant to examine accumulation of *in vivo* substrates and products.

## Experimental procedures

### Plant material and growth conditions

*Arabidopsis thaliana* ecotype Columbia plants were maintained in Sunshine Mix #1 soil at 22–24°C with 100–140 μmol m^−2^ s^−1^ light set for 16 h days. Mutant *impl2-3* and *impl2-4* plants were given exogenous histidine by watering with a 1 mM histidine solution every other day. Age-matched seeds after-ripened for 3 weeks at RT were used for all assays. Details of seed germination, root growth, and mutant selection are described in Supplemental Methods.

### Expression analyses

RNA was purified from soil grown plants, 3-d-old, and 7-d-old seedlings grown on 0.5× MS/1% sucrose-soaked filter paper under 16 h of light, as described in Donahue et al. (2010). Mature seeds, imbibed with water for 3 days at 4°C, were freeze-dried, followed by initial RNA extraction and LiCl precipitation (Vicente-Carbajosa and Carbonero, 2005). cDNA was synthesized from 2 μ g of RNA using Bio-Rad iScript cDNA synthesis kit, loaded into 96-well plates containing Sybr Green PCR MasterMix (Applied Biosystems) with gene-specific primers as described in Donahue et al. (2010).

### Constructs and imaging

IMP/IMPL ORFs without stop codons were amplified by PCR from Arabidopsis CS60000 cDNA. IMPs were cloned into pENTR/D-TOPO vector (Invitrogen), confirmed by sequencing, and recombined via the Gateway system (Invitrogen) using the manufacturer's protocol into destination vector pK7FWG2 (Karimi et al., 2002). The resulting vectors, IMP:GFP, IMPL1:GFP and IMPL2:GFP contain *Egfp* fused to the 3′ end of the cDNAs, under control of the 35S CaMV promoter, flanked by left border (LB), and right border (RB) and a plant Kanamycin resistance cassette. The constructs were transformed into *Agrobacterium tumefaciens* by cold shock and were used in stable transformation of wild-type plants and *impl2-3* and *impl2-4* mutant plants. Transformation of Arabidopsis was as described (Bechtold et al., 1993). Screening of plants and generation of transgenic plants for co-localization studies are described in Supplemental Methods online.

### LC-MS/MS analysis of histidine and histidinol

Tissues were harvested and immediately flash frozen in liquid nitrogen and were ground to fine powder in liquid nitrogen and lyophilized. Five mg of lyophilized seedlings and tissue samples were disrupted with glass beads and extracted with chloroform:10 mM HCl 1:1 (v/v) (1 ml final volume) and 40 μ M of norvaline was added to the aqueous phase as internal standard. The insoluble chloroform portion was removed by centrifugation. A portion of the (1:5 dilution) supernatant was dried and reconstituted in 200 μ l of 65% (0.1% formic acid and water) and 35% acetonitrile. The LC-MS/MS method used for histidine and histidinol analysis has been described previously (Gu et al., 2007) and modifications are described in the Supplemental Methods.

### Expression of recombinant protein and phosphatase activity assays

Plasmids containing the genes IMPL1 (At1g31190) and IMPL2 (At4g39120), designated pAtIMPL1H and pAtIMPL2H, respectively, were constructed as described in Torabinejad et al. (2009). The His 1-P substrate for IMPL2 was synthesized according to previous methods (Fujimoto and Naruse, 1967; Yoshikawa et al., 1967). The purity of the substrate was determined by Mass Spectrometry. In addition the absence of free phosphates was confirmed by a Malachite Green phosphate release assay. Phosphatase activity was determined by the inorganic phosphate quantification assay (Lanzetta et al., 1979) with minor modifications. Standard reaction conditions were 50 mM Tris-Cl, pH 7.5, 2 mM MgCl_2_, 0.4 mM substrate, and 112 ng of purified enzyme in a total reaction volume of 50 μl for IMPL2. Reaction conditions were 50 mM Tris, pH 9, 3 mM MgCl_2_, 0.4 mM substrate, and 452 ng of purified enzyme in a total reaction volume of 50 μl for IMPL1. Reactions were performed at room temperature (25°C) for 10 min, after which 800 μl of color reagent malachite green/ ammonium molybdate solution was added to terminate the reaction. The A_660_ was determined by a spectrophotometer. Control reactions without enzyme or without substrate were used to determine background phosphate levels, which were subtracted from experimental values. Enzyme-specific activity units are in μmol of phosphate. Protein concentrations were determined as described by Bradford Assay with bovine serum albumin as the standard. Data from kinetic experiments were analyzed with Kaleidograph software (version Mac; Synergy Software). Data were fit to the Michaelis-Menten equation *v* = V_max_ [S]/(K_m_ + [S]).

### Conflict of interest statement

The authors declare that the research was conducted in the absence of any commercial or financial relationships that could be construed as a potential conflict of interest.
